# A protein-rich meal provides beneficial glycemic and hormonal responses as compared to meals enriched in carbohydrate, fat or fiber, in individuals with or without type-2 diabetes

**DOI:** 10.3389/fnut.2024.1395745

**Published:** 2024-07-04

**Authors:** Neda Rajamand Ekberg, Sergiu-Bogdan Catrina, Peter Spégel

**Affiliations:** ^1^Department of Molecular Medicine and Surgery, Karolinska University Hospital, Karolinska Institutet, Stockholm, Sweden; ^2^Center for Diabetes, Academic Specialist Center, Stockholm, Sweden; ^3^Center for Analysis and Synthesis, Department of Chemistry, Lund University, Lund, Sweden

**Keywords:** alpha-cell, beta-cell, glucagon, insulin, insulin:glucagon ratio

## Abstract

**Introduction:**

Diet stands as a pivotal modifiable risk factor influencing weight gain and the onset of type-2 diabetes (T2D). This study delves into the variation in glucose and regulatory pancreatic hormone levels subsequent to the consumption of meals with differing macronutrient compositions.

**Methods:**

The cohort comprised 20 individuals diagnosed with T2D and 21 without diabetes. Participants underwent a cross-over design, consuming four isocaloric meals (600 kcal) enriched in carbohydrate, fiber, fat and protein. Plasma glucose, insulin and glucagon levels were measured at -30, and -5 min, followed by subsequent measurements every 30 min for 240 min post meal intake. Quantification of alterations in the postprandial state was accomplished through the incremental area under the curve (iAUC) and the incremental peak height for the insulin:glucagon ratio (IGR) and plasma glucose levels. The meal demonstrating the lowest responses across these variables was deemed the optimal meal.

**Results:**

Meals rich in protein and fat, and consequently low in carbohydrate, exhibited reduced incremental peak and iAUC for both glucose and the IGR in comparison to the other meals. While the protein-enriched meal neared optimal standards, it proved less efficient for individuals without T2D and possessing a low BMI, as well as in those with T2D and poor glycemic control.

**Conclusion:**

Our findings endorse the adoption of protein-enriched, low-carbohydrate meals to curtail the meal-induced anabolic hormonal response while averting excessive fluctuations in glucose levels.

## 1 Introduction

Obesity stands as a primary factor increasing the risk of developing type 2 diabetes (T2D). Multiple lines of evidence indicate that T2D can be reversed through weight loss, particularly when the disease duration is short (1, 2). Bariatric surgery, inducing rapid and sustained weight loss, leads to high rates of T2D remission (2), albeit not without associated complications (3). Another avenue toward weight loss and T2D remission involves low-calorie diets (LCDs) (1, 4). However, approximately 80% of individuals experience weight regain and T2D relapse, due to the challenge of sustaining reduced food intake (5).

T2D typically emerges in insulin resistant individuals who fail to compensate with appropriate hormone secretion (6). Consequently, defective insulin secretion underlies T2D development, characterized by the loss of first-phase insulin secretion. Initially, insulin levels in circulation are high but insufficient to counter insulin resistance (7). Hyperinsulinemia itself has been linked to weight gain (8), a significant driver in T2D progression. Nonetheless, the metabolic state is not solely regulated by insulin, as T2D can also be viewed as a condition of glucagon hypersecretion (9). To better describe this metabolic state, the insulin:glucagon ratio (IGR), introduced by Unger in the 1970’s (10), becomes crucial. The IGR predicts weight-changes following pharmaceutical interventions in individuals with T2D; drugs elevating the IGR leads to weight gain, while those exerting the opposite effect result in weight loss (11). However, the application of the IGR in dietary intervention studies has been less frequent (12).

Besides chronic hyperglycemia, postprandial glucose variability, measured as spike amplitude or the incremental area under the curve (iAUC), have been associated with the risk of developing diabetes complications (13, 14), presumably due to their capacity to induce metabolic memory, via, e.g., epigenetic mechanisms (13). Additionally, heightened postprandial insulin secretion has been associated with increased weight gain, particularly in insulin-sensitive individuals (15). Meal intake significantly influences our metabolism, and although transient, consuming three or more meals per day leads to postprandial phases occupying a significant portion of the day (16).

The objective of dietary intervention in individuals with or at risk of T2D should focus on minimizing postprandial variations in glucose levels and the IGR. This approach aims to mitigate the risk of developing adverse metabolic memory and an anabolic state promoting weight-gain, respectively. This study investigates the impact of varying meal compositions—protein, fat, carbohydrate and fiber—on postprandial glucose and hormone variability in individuals with and without T2D.

## 2 Materials and methods

### 2.1 Study population

The study encompassed 42 participants, with 21 diagnosed with T2D and 21 without diabetes (no diabetes, ND) (Table 1). Inclusion criteria comprised individuals aged between 20 and 75 years, having a body mass index (BMI) ranging from 25 to 33 kg/m^2^, and a T2D disease duration of more than 5 years for participants with T2D. Exclusion criteria included heart failure (NYHA class III & IV), renal failure (s-creatinine >200 μmoL/L), liver disease (ALAT >2 μKat/l), and current treatment with pioglitazone. The deliberate inclusion of a wide range of HbA1c levels and diabetes durations in the T2D sample aimed to capture the inherent heterogeneity of T2D. Detailed description of the inclusion and exclusion criteria have been provided previously (12). One participant diagnosed with T2D exhibited exaggerated insulin secretion (insulin >550 μU/mL) in response to all meals and was consequently excluded from the analysis. Comparatively, individuals with T2D displayed higher age (*p* = 0.022), BMI (*p* = 2.1 × 10^−6^) and HbA1c (*p* = 4.4 × 10^−7^) levels. Among the T2D group, treatment details were as follows: five received no treatment, 15 were on metformin, five on insulin, two on GLP-1, three on DPP4i, three on sulphonylurea, and one on acarbose. Insulin secretagogues and GLP-1 were discontinued the night before or in the morning preceding the intervention. The study was conducted according to the guidelines of the Declaration of Helsinki. The study protocol was approved by the Regional Ethical Review Board in Stockholm (2009/796-32, April 23 2009, ClinicalTrial.gov Identifier: NCT02544568). Written informed consent was obtained from all subjects involved in the study.

**Table 1:** Demographic characteristics of the populations.

	Type 2 diabetes^*^	Non diabetic^*^
Number	21	21
Women/men	11/10	12/9
Age (years)	64 (55–74)	52 (20–74)
BMI (kg/m^2^)	29 (25–33)	24 (19–32)
Diabetes duration (years)	11 (5–31)	
HbA1c (mmol/mol)	52 (40–84)	37 (29–42)

*Data are presented as mean (range).

### 2.2 Meal tolerance test

During the initial screening visit, participants underwent examinations, and fasting blood and urine samples were obtained. Subsequent to the screening phase and preceding the initial test meal, they were instructed to maintain a 3 day diary, and record capillary blood glucose measurements. They were also instructed to fast in the morning, before and 2 h post each meal, and prior to bedtime. On the day of the isocaloric lunches, participants were directed to consume a standardized breakfast (approximately 420–220 kcal, comprising 58% of energy from carbohydrates, 22% from protein, and 20% from fat) (Supplementary material) between 7:00–7:30 in the morning, around 4 h prior to the planned lunch. Two different matched breakfasts were offered to ensure compliance. Although we did not record which breakfast was chosen, it is expected that the variation in chosen breakfast alternatives was independent of the composition of the following lunch meal.

The study involved the consumption of four isocaloric meals (600 kcal each), differentiated by their enrichment in carbohydrate, protein, fat or fiber (Table 2) (17), in a crossover design with a minimum of one-month and two-months washout period for men and women, respectively. A longer washout period for women was implemented to mitigate the risk for anemia among menstruating women. The meal sequence was randomized before the study commencement. The meal compositions consisted of red meat, potatoes (boiled or French fries), and various vegetables/legumes, depending on the meal, and water to drink (Supplementary material). To replicate a typical Western diet and ensure the meal was enjoyable for most participants, the carbohydrate- and fiber-rich meals included sugar-sweetened berries. The carbohydrate-, fiber-, fat-, and protein-enriched diets contained 76.1, 72.5, 43.8, and 39.5 grams of carbohydrates, respectively, including 17.6 grams (10.9 E%) and 19.8 grams (12.3 E%) of added sugars in the carbohydrate- and fiber-enriched meals, respectively (Table 2). Hence, as a consequence of maintaining the diets isocaloric, the protein- and fat-rich meals contain much fewer carbohydrates and added sugars than the fiber- and carbohydrate-rich diets. All meals were prepared and served at a local restaurant within the Karolinska University Hospital. Study participants consumed a standardized breakfast at home (12), followed by the supervised ingestion of a test meal during lunchtime overseen by a research nurse. Blood samples were collected 30 and 5 min pre-meal intake (commencing at time 0) to establish the baseline metabolic state. Subsequently, samples were collected every 30 min from 30 min up to 240 min post-meal intake.

**Table 2:** Meal composition of macronutrients.

Macronutrients	High carbohydrate	High fiber	High fat	High protein
Carbohydrate (gram)	76.1	72.5	43.8	39.5
Carbohydrate (E%)	52	44.9	29	25.7
Protein (E%)	30.4	36.7	26.9	58.1
Fat (E%)	28.6	26.2	50	31.9
Sugars (gram)	35.8	37.9	6.5	7
Sugars (E%)	22.2	23.5	4.3	4.6
Of which added sugars (gram)	17.6	19.8	0	0
Of which added sugars (E%)	10.9	12.3	0	0
Starch (gram)	15.1	18.8	10.8	12.7
Starch (E%)	4.7	5.8	3.6	4.1

### 2.3 Laboratory assessments

The plasma glucose analysis followed standard procedures conducted at Karolinska University Hospital. HbA1c measurement employed the MonoS method (Unimate, Roche Diagnostics, Basel, Switzerland). Glucagon levels were assessed utilizing the RIA-kit GL-32 K (Millipore, MA), while insulin levels were determined using ELISA (DAKO, Agilent Technologies, Glostrup, Denmark).

### 2.4 Statistical analyses

All analyses were conducted using R (version 4.2.3). Normality was assessed using the Shapiro–Wilk test (shapiro.test, stats). Variables that were non-normally distributed were log2-transformed. The incremental area under the curve (iAUC) was calculated using the trapezoid rule (auc, flux), with the baseline level defined as the average of the variable determined at −5 and −30 min of the meal-tolerance test. The incremental peak response was characterized as the difference between maximum and minimum glucose or hormone levels during the postprandial phase. Data were subjected to analysis employing linear mixed-effects models (lmer, lme4) followed by a false-discovery rate adjusted Tukey test *post hoc* (glht, multcomp). Differences between glycemic groups (ND or T2D) were evaluated using the Student’s *t*-test (*t*.test, stats). Associations were examined using linear models (lm, stats). Euclidian distances were computed using the dist function (stats) on standardized data (unit variance-scaled and mean centered; scale, base). Visualization was performed using ggplot (ggplot2). Data are presented as mean ± standard deviation. A significance level of *p* < 0.05 was considered statistically significant.

## 3 Results

### 3.1 Postprandial glucose

Fasting glucose levels showed no variance between meals but were notably elevated (*p* = 5.7 × 10^−5^) in subjects diagnosed with T2D (6.59 ± 1.38 mM) compared to ND individuals (5.00 ± 0.39 mM), with a slight elevation in individuals under metformin treatment (*p* = 0.030) (glucose trajectories of all study participants are shown in Supplementary Figure S1A). Moreover, in T2D subjects, the time taken to reach peak glucose levels was delayed compared to ND subjects, shifting from 66 ± 61 min to 92 ± 43 min (*p* = 0.0039) for the carbohydrate rich meal, from 66 ± 49 min to 94 ± 72 min (*p* = 0.0057) for the fiber rich meal, and from 76 ± 49 min to 89 ± 89 min (*p* = 0.0052) for the protein-rich meal. The timing of peak glucose remained unaffected by hypoglycemic treatment (insulin or metformin).

**Figure 1:**
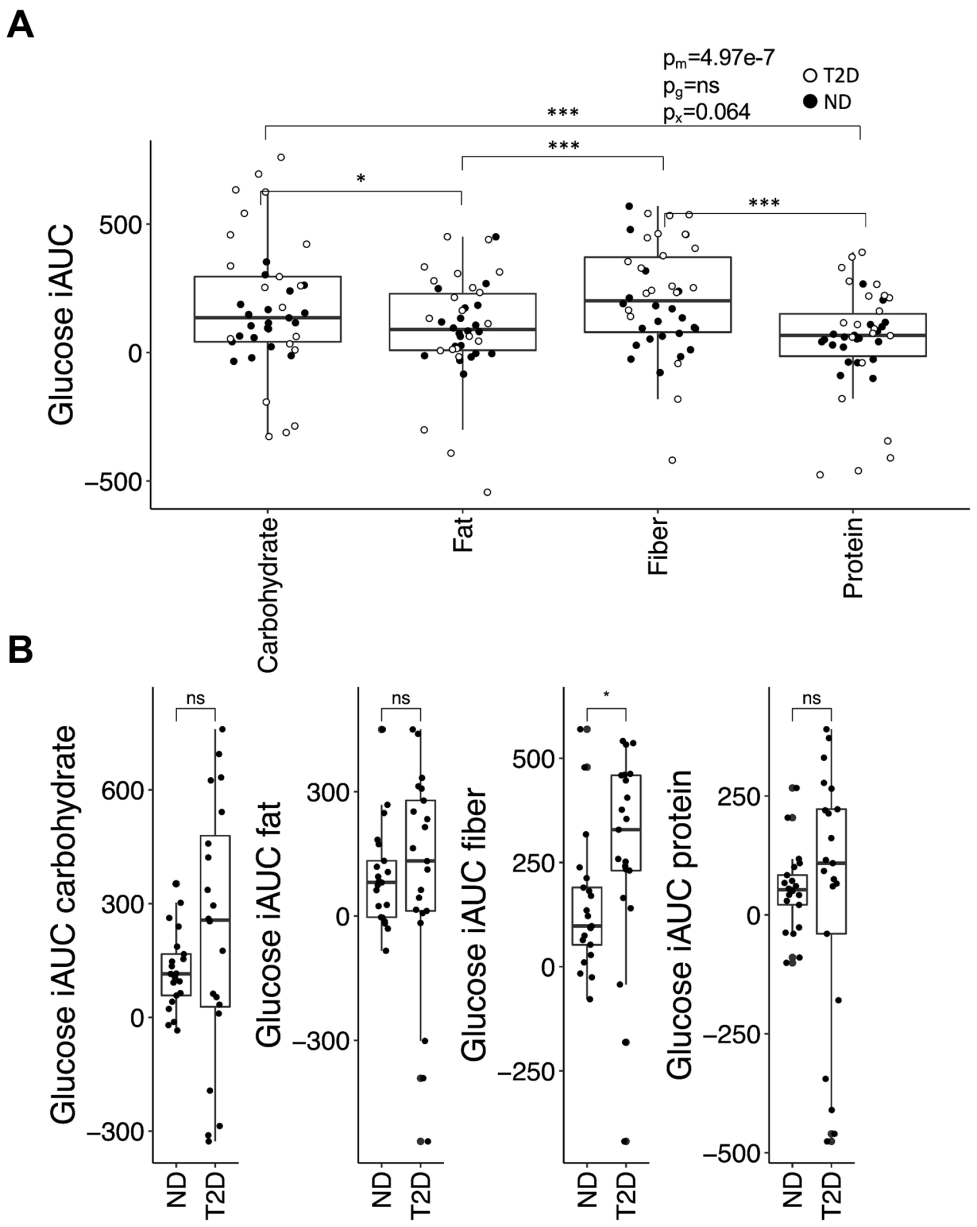
The glucose iAUC in the postprandial phase. **(A)** The iAUC for glucose after consumption of meals enriched in carbohydrates, fiber, fat or protein. **(B)** Differences in iAUC for glucose between individuals with type-2 diabetes (T2D) and without T2D (no diabetes; ND). Differences between groups were assessed by linear mixed-effects models with the Tukey’s test *post hoc* **(A)** or the Student’s *t*-test **(B)**. *p < 0.05 and ***p < 0.001; ns, not significant. *p*-values are reported for the effect of meal (pm), glycemic group (ND or T2D; pg) and their interaction (px) in **(A)**.

Postprandial glucose iAUC was contingent upon meal composition (*p* = 2.9 × 10^−7^), with higher values observed for carbohydrate- and fiber-rich meals in comparison to fat- or protein-enriched meals (Figure 1A). Additionally, there was a borderline significant interaction between meal type and glycemic state (ND or T2D) (*p* = 0.064). This interaction stemmed from a higher iAUC in T2D subjects than ND subjects following the consumption of the fiber-rich meal (*p* = 0.045); no significant differences in iAUC for glucose were discerned between T2D and ND subjects for the other meal types (Figure 1B). These differences in iAUC persisted even after adjusting for metformin and insulin use.

**Figure 2:**
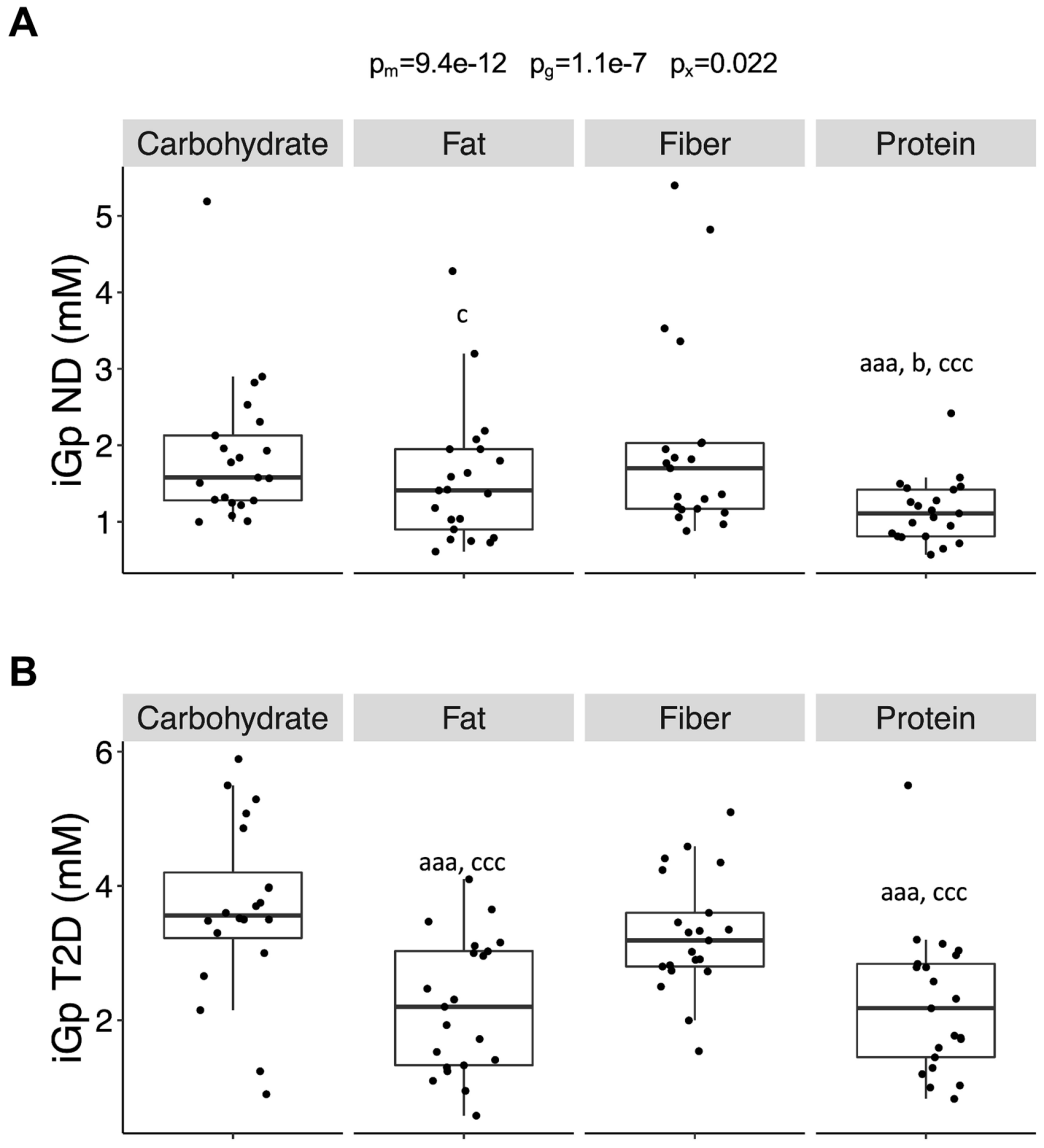
The incremental peak glucose in the postprandial phase. **(A)** The incremental glucose peak (iGp) in individuals without diabetes (no diabetes; ND) and **(B)** in individuals with type-2 diabetes (T2D) after consumption of the four different meals. Differences between groups were first assessed by linear mixed-effects models and *p*-values reported as described in Figure 1. Data were then stratified by glycemic group (ND or T2D) and differences between meals assessed by linear mixed-effects models with the Tukey’s test *post hoc*. Significance is given by lower case letters: a versus carbohydrate, b versus fat, and c versus fiber. Aaa/ccc, *p* < 0.001, c/b, *p* < 0.05.

The incremental glucose peak (iGp) was influenced by both meal composition (*p* = 9.4 × 10^−12^) and glycemic status (*p* = 1.1 × 10^−7^), alongside their interaction (*p* = 0.022). These effects remained irrespective of whether individuals with T2D were treated with insulin or metformin. iGp was consistently higher in T2D subjects compared to ND subjects across all meal compositions (carbohydrate- (*p* = 2.0 × 10^−5^), fiber- (*p* = 0.00043), protein- (*p* = 0.00021), and fat-rich (*p* = 0.030) meals). The relative increase in iGp among T2D compared to ND subjects was 74 ± 0.73%, showing no variance across meals. iGp for the protein-rich meal was lower compared to carbohydrate- and fiber-rich meals in both T2D and ND subjects (*p* < 0.001, Figure 2). T2D individuals exhibited lower iGp for the fat-rich meal compared to carbohydrate- and fiber-rich meals (*p* < 0.001), while in ND subjects, iGp after the fat-rich meal was slightly lower than after the fiber-rich meal (*p* = 0.035) but not after the carbohydrate-rich meal.

### 3.2 Postprandial insulin

Fasting insulin levels exhibited no significant differences between meals but were approximately twofold higher in T2D compared to ND subjects (*p* = 0.00045) (insulin trajectories of all study participants are shown in Supplementary Figure S1B). The time taken to reach peak insulin levels was delayed in T2D subjects compared to ND subjects for both the carbohydrate- (79 ± 65 min for T2D and 43 ± 15 min for ND; *p* = 0.00030) and the fiber-rich meals (74 ± 59 min for T2D and 57 ± 25 min for ND; *p* = 0.0083). Fasting insulin and the timing of peak insulin remained independent of hypoglycemic treatment (insulin or metformin).

The iAUC for insulin was contingent upon meal composition (*p* = 6.6 × 10^−9^) but showed no dependency on glycemic state. iAUC was notably higher following the consumption of carbohydrate- and fiber-rich meals in both T2D (*p* < 0.05) and ND subjects (*p* < 0.01). These effects persisted even after adjusting for insulin and metformin use, with slightly lower iAUC observed in individuals under metformin treatment (*p* = 0.014).

The incremental insulin peak (iIp) was influenced by meal composition (*p* = 1.5 × 10^−7^) but remained independent of glycemic group and hypoglycemic treatment (insulin or metformin). iIp was lower for the fat- (*p* = 1.7 × 10^−6^ vs. carbohydrate; *p* = 0.00014 vs. fiber) and protein-rich (*p* = 0.00014 vs. carbohydrate; *p* = 0.0058 vs. fiber) meals, compared to the other two meals.

### 3.3 Postprandial glucagon

Fasting glucagon levels did not exhibit variance between meals but displayed a trend towards being approximately 35% higher in T2D compared to ND subjects (*p* = 0.093) (glucagon trajectories of all study participants are shown in Supplementary Figure S1C). The timing of peak glucagon was later for the protein-rich meal compared to the carbohydrate- (*p* = 0.047) and fiber-rich (*p* = 0.026) meals. This timing difference was independent of glycemic group and hypoglycemic treatment (insulin or metformin).

The iAUC for glucagon was contingent upon meal composition (*p* = 4.8 × 10^−12^) but did not rely on glycemic state. iAUC was notably higher after the intake of the protein-rich-meal compared to all other meals in both subjects with T2D (*p* < 7 × 10^−6^) and ND subjects (*p* < 0.001). Neither insulin nor metformin use exerted an impact on the glucagon iAUC.

The incremental glucagon peak (iGlp) was influenced by both meal composition (*p* = 0.0016) and glycemic state (*p* = 0.0017). These differences persisted even after adjusting for metformin and insulin use. iGlp was higher in subjects with T2D as compared to ND subjects for the carbohydrate- (*p* = 0.026), fiber- (*p* = 0.0093) and fat-rich (*p* = 0.017) meals, and showed a tendency to be higher for the protein-rich meal (*p* = 0.057). In ND subjects, iGlp was higher for the protein-rich meal compared to the carbohydrate- (*p* = 0.0096), fiber- (*p* = 0.014), and fat-rich meals (*p* = 0.0062); however, no difference in iGlp between meals were observed in subjects with T2D.

### 3.4 Postprandial insulin:glucagon ratio

Fasting IGR did not demonstrate variance between meals but was approximately twice as high (*p* = 0.0026) in T2D compared to ND subjects (IGR trajectories of all study participants are shown in Supplementary Figure S1D). Fasting IGR remained unaffected by metformin and insulin treatment. The timing of peak IGR did not differ between meals and glycemic groups; however, individuals on insulin treatment displayed a slightly earlier peak IGR (*p* = 0.032).

**Figure 3:**
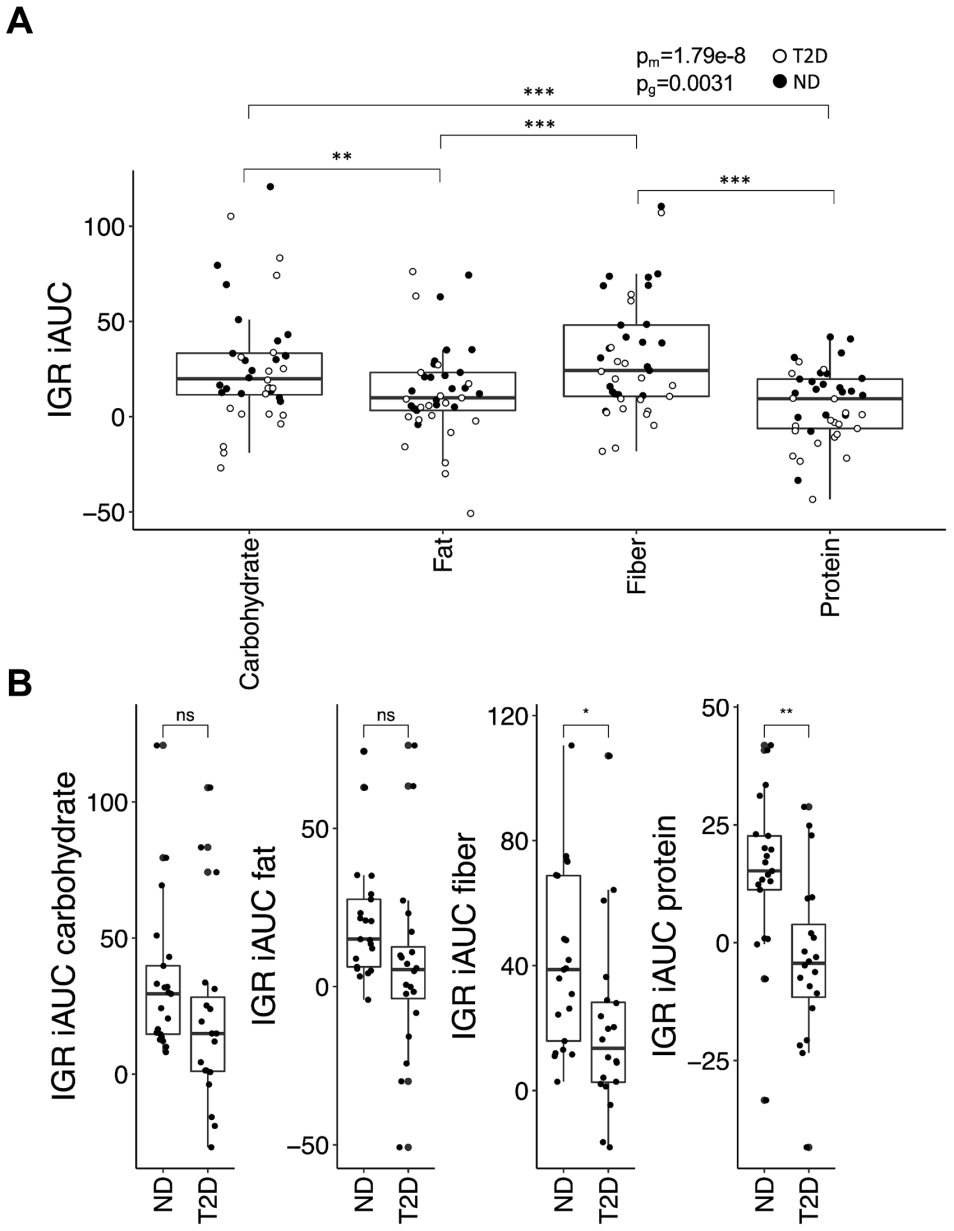
The insulin:glucagon ratio (IGR) iAUC in the postprandial phase. **(A)** The iAUC for IGR after consumption of the four different meals and **(B)** differences in the meal response between individuals with type-2 diabetes (T2D) and without type-2 diabetes (no diabetes; ND). Data were analyzed and presented as outlined in Figure 1.

The iAUC for IGR was contingent upon both meal composition (*p* = 2.5 × 10^−8^) and glycemic group (*p* = 0.0031) (Figure 3A). In ND subjects, the carbohydrate-rich meal generated a higher iAUC for IGR than the protein-rich meal (*p* = 0.0060) and tended to produce a higher iAUC compared to the fat-rich meal (*p* = 0.065). Similarly, in these subjects, the iAUC for IGR was greater after the fiber-rich meal than after both the protein- (*p* = 0.00018) and the fat-rich meals (*p* = 0.0038). A similar trend was observed in subjects with T2D, with the carbohydrate- and fiber-rich meals yielding higher iAUC for IGR than the protein- (*p* = 0.0017 and *p* = 0.0017, respectively) and fat-rich meals (*p* = 0.065 and *p* = 0.065, respectively). The iAUC for IGR was greater in ND subjects than subjects with T2D for the protein- (*p* = 0.0015) and fiber-rich meals (*p* = 0.024), with a borderline significant difference for the fat-rich meal (*p* = 0.065) (Figure 3B). Associations with iAUC remained significant after adjusting for insulin and metformin use, although metformin (*p* = 0.0002) and insulin (*p* = 0.027) were associated with a lower iAUC for IGR.

**Figure 4:**
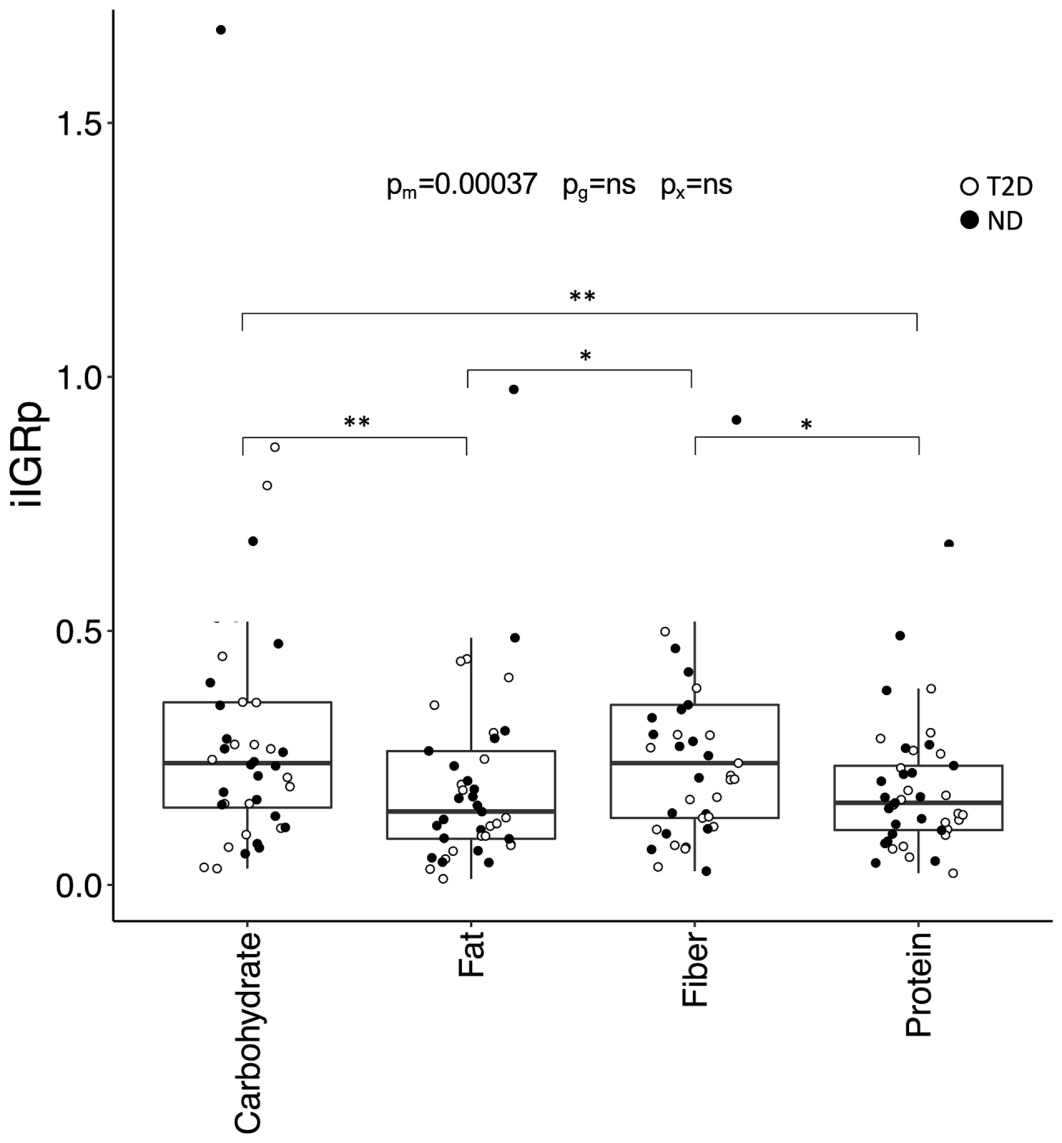
The incremental peak insulin:glucagon ratio (IGR) in the postprandial phase. Incremental IGR peak (iIGRp) after consumption of the four test meals. Data are presented and analyzed as described in Figure 2.

The incremental IGR peak (iIGRp) was influenced by meal composition (*p* = 0.00037) and remained independent of glycemic group. Both the carbohydrate- and fiber-rich meals elicited a higher iIGRp than the fat- (*p* = 0.0060 vs. carbohydrate; *p* = 0.039 vs. fiber) and protein-rich (*p* = 0.0017 vs. carbohydrate; *p* = 0.011 vs. fiber) meals (Figure 4). These differences persisted even after adjusting for insulin and metformin use, although iIGRp was marginally lower in individuals on metformin (*p* = 0.035).

### 3.5 Defining the optimal macronutrient

**Figure 5:**
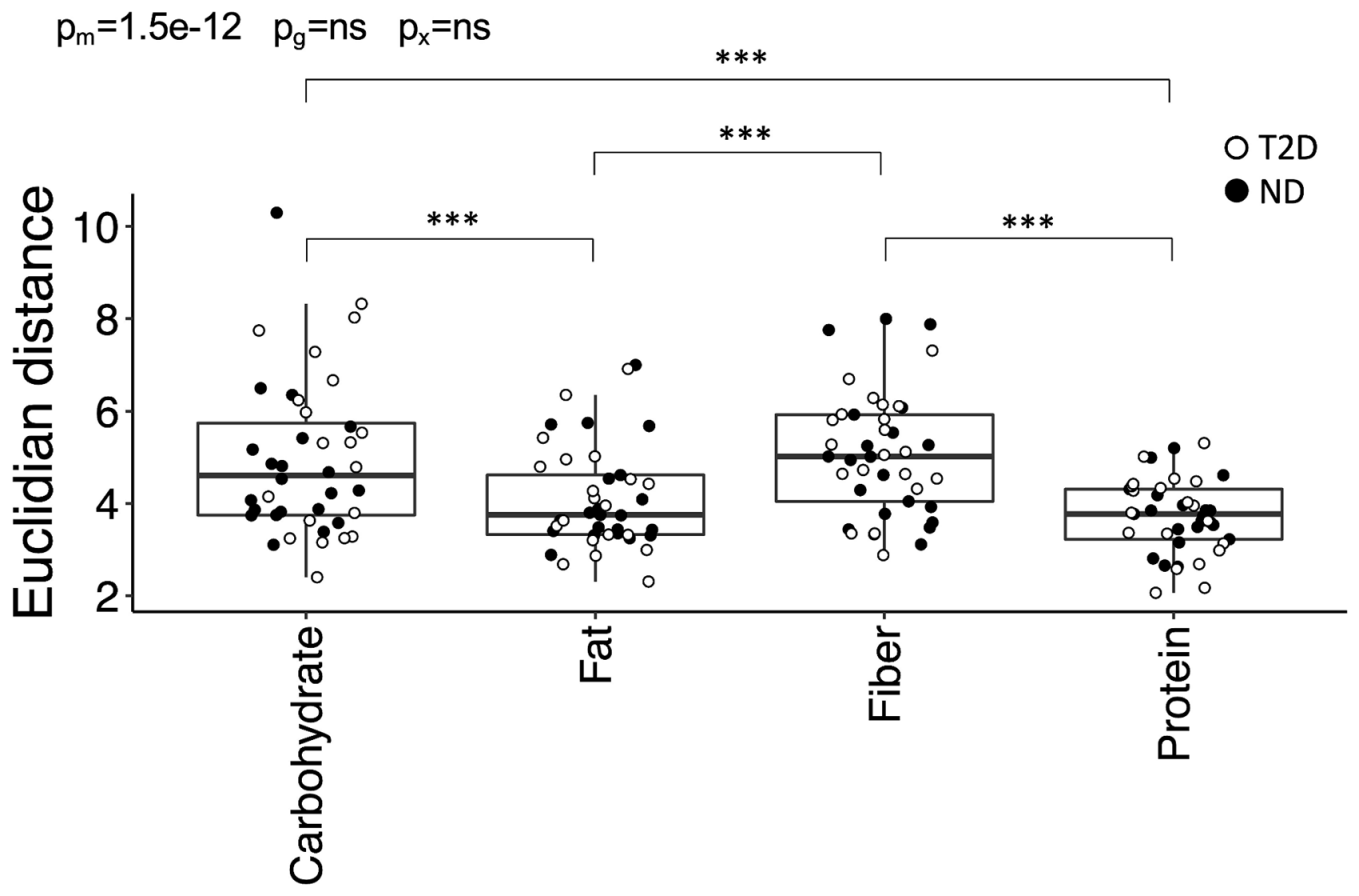
The distances between the carbohydrate-, fiber-, fat-, and protein-rich meals and the optimal meal in Euclidian space. Distances between the four meals and the optimum meal, as defined by the lowest recorded iAUC and incremental peak for glucose and the iAUC. Data are presented and analyzed as outlined in Figure 1A.

The determination of the optimal meal macronutrient composition concerning postprandial glycemic and hormonal regulation was based on identifying the meal that yielded the lowest values for iIGRp, iGp, iAUC for glucose and iAUC for the IGR. Determining the specific importance of these elements in preserving the health status remain uncertain. To address this, all variables underwent standardization, and the Euclidian distance between all data points and the most favorable meal in the Euclidian space was calculated. The Euclidian distance was contingent upon meal composition (*p* = 9.2 × 10^−13^) but demonstrated no dependence on glycemic state. Specifically, the Euclidian distance for the fat- (*p* = 9.2 × 10^−5^ vs. carbohydrate; *p* = 6.9 × 10^−6^ vs. fiber) and protein-rich (*p* = 1.4 × 10^−8^ vs. carbohydrate; *p* = 4.7 × 10^−10^ vs. fiber) meals was smaller compared to the other two meals (Figure 5). These outcomes persisted even after further adjustment for metformin and insulin use, revealing an association between metformin and a reduced distance (*p* = 0.028).

### 3.6 Individual variation in metabolic response

We conducted an investigation to determine whether differences in BMI, HbA1c, sex and diabetes duration, could account for the observed individual variations post-meal. As anticipated, variation in HbA1c was more pronounced in T2D compared to ND subjects (*p* = 1.7 × 10^−5^). Additionally, variation in age was notably smaller among individuals with T2D (*p* = 5.5 × 10^−7^), while variation in BMI remained independent of glycemic group. There was no association between age and diabetes duration among individuals with T2D (*p* = 0.44 for the regression coefficient). In individuals with T2D, diabetes duration exhibited a negative association with the Euclidian distance following the intake of the fiber- (*p* = 0.00066), fat- (*p* = 0.0084) and carbohydrate-rich meal (*p* = 0.0045) (Supplementary Figure S2). These associations remained significant even after further adjustment for insulin and metformin use. In ND subjects, BMI showed a positive association with the Euclidian distance (*p* = 0.0031) following the intake of the carbohydrate-rich meal (Supplementary Figure S3). No associations were found with HbA1c.

**Figure 6:**
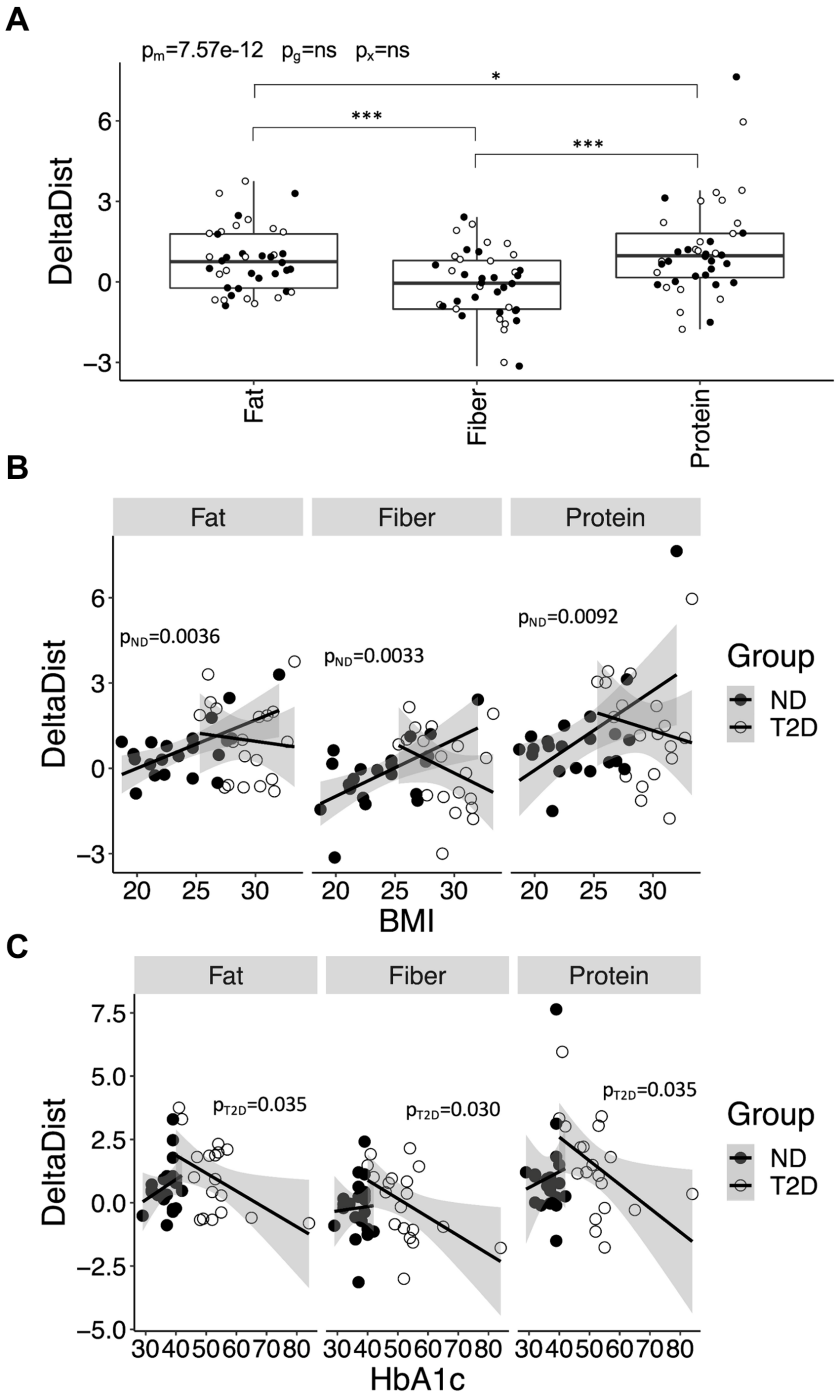
Improvements in postprandial glycemic and hormonal regulation for meals enriched in fiber, fat and protein, relative to carbohydrate, and its variation with health-related variables. **(A)** The improvement in postprandial glucose and hormone regulation for the fat-, fiber-, and protein-rich meals, relative the carbohydrate-rich meal, in Euclidian space (DeltaDist). **(B)** Associations between DeltaDist and BMI in individuals with type-2 diabetes (T2D) and without T2D (no diabetes; ND). **(C)** Associations between DeltaDist and HbA1c in ND and T2D. Data in **(A)** are presented and analyzed as outlined in Figure 1A. Data in **(B,C)** were stratified for glycemic group (ND or T2D) and analyzed using linear regression. *p*-values are provided for ND (pND) and T2D (pT2D).

We further explored whether certain individuals were more likely to benefit from switching from a carbohydrate-rich meal to a meal enriched in other nutrients. To evaluate this, we calculated the difference in Euclidian distances for the fat-, fiber-, and protein-rich meals relative to the carbohydrate rich meal. These analyses indicated that the gain was greater for the protein-rich meal compared to the fiber- (*p* = 5.7 × 10^−12^) and fat-rich (*p* = 0.037) meals, irrespective of whether the individual had T2D or not, or whether they were being treated with metformin or insulin (Figure 6A). Subsequently, we examined the association between the difference in Euclidian distances with HbA1c, BMI and diabetes duration. Among individuals without diabetes, those with a higher BMI appeared to benefit more from switching from carbohydrates to fiber (*p* = 0.0033), protein (*p* = 0.0092) and fat (*p* = 0.0036) (Figure 6B). Conversely, among those diagnosed with T2D, the benefit derived from switching from carbohydrates to fiber (*p* = 0.030), protein (*p* = 0.035) or fat (*p* = 0.035) diminished with increasing level of HbA1c (Figure 6C). Sex did not exhibit influence, although a longer duration of T2D tended to associate with a lower gain from a protein-rich meal (*p* = 0.064) (Supplementary Figure S4). Associations between HbA1c and diabetes duration with the difference in Euclidian distance persisted even after further adjustments for insulin and metformin use.

## 4 Discussion

The primary objective of this study was to investigate the impact of various macronutrient compositions in complex solid meals on postprandial blood glucose and its regulatory pancreatic hormones. Previous research primarily focused on liquid meals, potentially leading to simplified physiological response (18). In addition to the three main macronutrients (carbohydrates, protein, and fat), we included a fiber-rich meal to offer a more comprehensive understanding of the postprandial response to essential food components.

Our analyses focused on glucose, closely associated with T2D, and IGR, pertinent to metabolic status and changes in body weight (11). These variables were quantified as the iAUC, differentiating the meal-associated response from fasting glucose and IGR differences between ND and T2D. We also considered incremental peak glucose and IGR responses to gauge postprandial metabolic variability.

Overall, protein- and fat-rich meals produced lower postprandial response in glucose and IGR compared to fiber- and carbohydrate-rich meals. While this aligns with expectations for postprandial glucose measurements, hormonal response is harder to predict. Various amino acids either potentiate or stimulate secretion of insulin (19) and glucagon (20), as well as hormones such as GLP-1 (21) and somatostatin (22), which modulate secretion of the former islet hormones. Additionally, fatty acids impact on the secretion of these hormones (12).

Notably, the glucose iAUC was higher in subjects with T2D than in ND subjects after the fiber-rich meal, unlike any of the other meals. Similarly, the fiber-rich meal demonstrated a reduced iAUC for IGR in T2D compared to ND. Dietary fibers are typically known to improve glycemic control by delaying gastric emptying, reducing the degradation rate of amylopectin, and slowing the uptake of resulting sugars (23). However, it remains unclear why dietary fibers resulted in a higher iAUC in subjects with T2D. The observed delay in peak glucose and insulin among subjects with T2D following consumption of carbohydrate- and fiber-rich meals confirms a slower gastric emptying rate in comparison to ND subjects (24). In T2D, several hormones that regulate gastric emptying (incretins, duodenal, pancreatic and orexigenic hormones (25), including peptide tyrosine-tyrosine (PYY) and ghrelin) are altered, potentially explaining our findings (26, 27). Another explanation could be attributed to the type of fibers used in the studies. Most previous research on the metabolic consequences of fiber intake utilized fiber-preloads or dietary regimens with fiber-enriched foods in healthy subjects, rather than investigating postprandial responses to a single meal in individuals with T2D (28). In our study, we utilized legumes as a source of fiber, differing from the super-physiological concentrations of specific fibers used in the majority of previous studies (28).

We observed a significantly lower IGR iAUC in response to the protein-rich meal in subjects with T2D compared to ND subjects, despite similar glucose iAUCs. This suggests that a protein-rich meal induces a less pronounced anabolic state in subjects with T2D without compromising postprandial glycaemia. The underlying reason for this observation remains unclear but might relate to alterations in gastric emptying and glycemia, processes regulated by amino acid-responsive hormones (29).

Only minor differences were noted in the meal-dependent incremental peak glucose and IGR response between T2D and ND subjects. However, the differences in iGP between meals was more pronounced in T2D compared to ND subjects, emphasizing poorer glycemic control in this group.

Up to this point, the study’s findings favor protein- and fat-rich meals over the carbohydrate- and fiber-rich meals. This trend applies to both individuals with T2D and without, although there are noticeable differences in how they respond to the four meal types. To pinpoint the most beneficial meal in terms of postprandial effects, we assessed the distance between all meals and the optimal meal. The optimal meal was identified as the one leading to the lowest incremental peak and iUAC for glucose and the IGR. These analyses reaffirmed the advantageous postprandial effects of protein- and fat-rich meals compared to the other two options. The distance showed no variance between T2D and ND individuals and remained unaffected by insulin and metformin treatment. However, it generally increased with prolonged diabetes duration, aligning with the recognized decline in metabolic control over time (30). In ND subjects, a positive association between the distance and BMI was noted for the carbohydrate-rich meal, in line with the anticipated rise in insulin resistance with increased BMI (31).

Finally, the study delved into the potential benefits for study participants when transitioning from a carbohydrate-rich meal to ones with higher concentration of other macronutrients. These analyses demonstrated that the advantage was more pronounced with the protein-rich meal compared to the other two meal types, irrespective of hypoglycemic treatment, in both ND and T2D subjects. However, not all individuals experienced the same level of benefit. In participants with T2D, the benefits associated with a transition from carbohydrates to other macronutrients declined as HbA1c levels increased, implying that altering the macronutrient composition of meals may be less effective for individuals with poorly controlled diabetes. This may be due to a proportional decrease of the insulin response or secondary to the “toxic effect” that high glucose levels have on insulin sensitivity. Nonetheless, advocating for increased protein intake in individuals with T2D should be approached cautiously due to the heightened prevalence of kidney disease in this population (32). Some evidence implies that high-protein diets could potentially exacerbate renal function due to intraglomerular hypertension (33). However, a systematic review examining protein diets in T2D did not conclusively confirm this effect (34). Additionally, it is crucial to note that individuals at risk of developing dyslipidemia should steer clear of a diet high in fat (35), although the composition of fats may influence this risk (36). The disparities observed between the T2D and ND groups regarding the benefits of transitioning from carbohydrates to other macronutrients may, to some extent, be influenced by the greater variation in HbA1c levels among individuals diagnosed with T2D.

Interestingly, the advantage gained from transitioning from a carbohydrate-rich meal to meals enriched with other macronutrients amplified with higher BMI in ND subjects. This escalation likely stems from heightened insulin secretion in response to carbohydrates among individuals with insulin-resistance and obesity (31). Therefore, in individuals without T2D, the benefit is more significant for those at the highest risk of developing T2D and requiring weight loss.

We did not evaluate effect of age on hormonal and glycemic responses, as the age-range was too narrow in the T2D group. One limitation of this study is the examination limited to the metabolic effects of a single meal, making it challenging to draw conclusions about the long-term impact of dietary habit changes. However, our diet’s long-term consequences likely result from the cumulative effects of repeated meal intake, given that the fasted state is unlikely to stimulate our metabolism to the same extent. Supporting this notion, consistent with the findings of this study indicating an increased catabolic state in response to the protein-rich meal with increasing BMI, previous studies demonstrated that women with obesity tend to lose more lean mass than women without obesity when on a protein-enriched, energy-deficient diet (37). Importantly, both groups experienced lower lean body mass losses compared to a matched low-protein diet (37). Similarly, this study’s results regarding the fat-rich meal align with previous research showing that individuals with obesity tend to lose more weight in response to high-fat diet compared to leaner individuals (38). Another limitation in our study was the lack of control over the sequence in which meal components were consumed. Multiple independent pieces of evidence suggest that the order of macronutrient consumption in a single meal impacts the metabolic response, likely due to its effect on gastric emptying (39). Yet, implementing strategic eating may pose challenges in everyday life. Moreover, our study utilized a restricted set of food components to control the meal’s protein content. The metabolic effect of protein intake, however, relies on the peptides and amino acids formed during ingestion. In line with this, and somewhat surprising given the result of the present study in which animal-derived protein was used, epidemiological studies have found animal-derived protein to increase the risk of developing T2D in women with obesity, whereas no effect was observed in men or in any of the sexes for plant-derived protein (40). Nonetheless, a systematic review examining protein diets in T2D did not find sufficient evidence to recommend plant protein over animal protein (34). One more limitation of the study is that participants could choose between two different breakfast meals to improve compliance. Despite the macronutrient content, including fiber content, of these meals being carefully matched, we cannot rule out contributions from a second-meal effect on our results. However, within our relatively small sample, we do not expect to detect any significant differences in breakfast choices between investigated groups, and the second-meal effect is hence only expected to introduce random variation.

Finally, it must be emphasized that meals enriched with protein and fat also contained almost half the carbohydrates, particularly lower levels of added sugars, which may drive many of our observations. Although the amount of added sugar may seem high, reaching up to 12.3 E% in this study, it mirrors a Western diet where added sugars contribute up to 19% of total energy intake in some age categories (41). Our results align with multiple long-term studies showing that low-carbohydrate, high-protein, or high-fat diets elicit several beneficial metabolic effects, including increased energy expenditure and weight loss, and reduced insulin resistance, fasting glucose, and HbA1c, compared to high-carbohydrate diets (42–44). Hence, while it is difficult to draw exact conclusions on the impact of the various macronutrients on meal responses in the present study, the findings do provide some direction on how to prepare realistic meals that promote beneficial glycemic and hormonal responses.

## 5 Conclusion

The study outcomes demonstrate advantageous postprandial effects following the consumption of a protein-rich, carbohydrate-poor meal compared to meals rich in carbohydrates, fiber or fat. Specifically, individuals with T2D and a well-controlled glycaemia and those without T2D but with a high BMI exhibited reduced levels and variability of glucose and hormones after consuming a protein-rich, carbohydrate-poor meal.

## Data availability statement

The original contributions presented in the study are included in the article/Supplementary material, further inquiries can be directed to the corresponding authors.

## Ethics statement

The studies involving humans were approved by the Regional Ethical Review Board in Stockholm. The studies were conducted in accordance with the local legislation and institutional requirements. The participants provided their written informed consent to participate in this study.

## Author contributions

NE: Conceptualization, Data curation, Funding acquisition, Investigation, Methodology, Project administration, Resources, Writing – review & editing. S-BC: Funding acquisition, Investigation, Methodology, Project administration, Resources, Writing – review & editing. PS: Conceptualization, Data curation, Formal analysis, Funding acquisition, Investigation, Methodology, Project administration, Visualization, Writing – original draft, Writing – review & editing.
