# Supplementary material for: A protein-rich meal provides beneficial glycemic and hormonal responses as compared to meals enriched in carbohydrate, fat or fiber, in individuals with or without type-2 diabetes
Source: Front Nutr. 2024 Jul 4;11:1395745. doi: 10.3389/fnut.2024.1395745 (PMC11257041; doi:10.3389/fnut.2024.1395745)
Supplement: Supplementary file 1 [file Data_Sheet_1.docx]

Supplementary Material

A protein-rich meal provides beneficial glycemic and hormonal responses as compared to meals enriched in carbohydrate, fat or fiber, in individuals with or without type-2 diabetes

Neda Rajamand Ekberg^1,2^, Sergiu-Bogdan Catrina^1,2^, and Peter Spégel^3, *^

^1^Department of Molecular Medicine and Surgery, Karolinska University Hospital, Karolinska Institutet, Stockholm, Sweden

^2^Center for diabetes, Academic Specialist Center, Stockholm, Sweden

^3^Center for Analysis and Synthesis, Department of Chemistry, Lund University, Lund, Sweden.

*** Correspondence:** Peter Spégel: [peter.spegel@chem.lu.se](mailto:peter.spegel@chem.lu.se)

**Supplement Pages**

Standardized breakfast 2

Carbohydrate-rich meal 3

Fiber-rich meal 4

Protein-rich meal 5

Fat-rich meal 6

Supplemental Figure S1 7

Supplemental Figure S2 8

Supplemental Figure S3 9

Supplemental Figure S4 10

**Standardized breakfast in the morning before Isocaloric meals**

Participants should have breakfast between 7:00 – 7:30 in the morning (4 hours before the planned lunch).

***Breakfast Option 1:***

Oatmeal with semi-skimmed milk, 1 half roll with light margarine, smoked ham and tomato, orange, coffee

Energy: 450 kcal (431)

Protein: 21% of energy

Fat: 19% of energy, including 7% saturated, 7% monounsaturated, and 4% polyunsaturated

Carbohydrate: 60% of energy, 63 g

Fibre: 10 g

***Breakfast Option 2:***

Caffé latte with 3 dl skimmed milk, 1 slice of rye bread with light margarine and smoked ham, 1 slice of rye bread with Becel and 17% cheese, tomato, orange

Energy: 400 kcal (419)

Protein: 24% of energy

Fat: 20% of energy, including 10% saturated, 6% monounsaturated, and 3% polyunsaturated

Carbohydrate: 56% of energy, 58 g

Fibre: 8 g

**Carbohydrate-rich meal**

Main dish: **Oven-Roasted Roast Beef with Stir-Fried Vegetables and Boiled Potatoes**

Dessert: **Sugared Blueberries and Raspberries with Whipped Cream**

Ingredients

Sauce: Soy sauce: 6 g, rapeseed oil: 3 g, (optional garlic), a pinch of sambal oelek, a pinch of tomato paste, 0.3 tsp freshly grated ginger, salt, pepper

Rapeseed oil: 5 g

Frozen corn kernels: 50 g

Fresh broccoli: 75 g

Carrot: 50 g

Yellow/red onion: 30 g

Leek: 30 g

Oven-roasted roast beef: 75 g

Potatoes, peeled, boiled: 160 g

Sugared blueberries, frozen: 80 g

Sugared strawberries, frozen: 80 g

Unwhipped whipping cream: 20 g

Preparation

Mix and combine the sauce ingredients.

Slice the vegetables, stir-fry them in the oil until they are evenly soft.

Drizzle the sauce over the stir-fried vegetables.

**Fiber-rich meal**

Main dish: **Oven-roasted Roast Beef with Stir-fried Vegetables, beans and Boiled Potatoes**

Dessert: **Sugared Blueberries and Raspberries with Whipped Cream**

Ingredients

Sauce: Soy sauce: 6 g, rapeseed oil: 3 g, (optional garlic), a pinch of sambal oelek, a pinch of tomato paste, 0.3 tsp freshly grated ginger, salt, pepper

Rapeseed oil: 7 g

Fresh broccoli: 75 g

Carrot: 50 g

Yellow/red onion: 30 g

Leek: 30 g

Red beans: 70 g

Canned haricots verts: 70 g

Oven-roasted roast beef: 80 g

Potatoes, peeled, boiled: 100 g

Sugared blueberries, frozen: 75 g

Sugared strawberries, frozen: 75 g

15% whipped cream: 30 g

Preparation

Mix and combine the sauce ingredients.

Slice the vegetables, stir-fry them in oil until evenly soft. Stir in the beans.

Drizzle the sauce over the vegetables.

**Protein-rich meal**

Starter: **Avocado with Shrimp**

Main dish: **Oven-roasted Roast Beef with Stir-fried Vegetables and Boiled Potatoes**

Ingredients

Peeled shrimp: 50 g

Avocado: 50 g

Sauce: Soy sauce: 5 g, rapeseed oil: 2 g, (optional garlic), a pinch of sambal oelek, a pinch of tomato paste, 0.3 tsp freshly grated ginger, salt, pepper

Rapeseed oil: 3 g

Frozen corn kernels: 30 g

Fresh broccoli: 100 g

Yellow/red onion: 35 g

Leek: 35 g

Oven-roasted roast beef: 150 g

Potatoes, peeled, boiled: 150 g

Preparation

Mix and combine the sauce ingredients.

Slice the vegetables, stir-fry them in the oil until they are evenly soft.

Drizzle the sauce over the stir-fried vegetables.

**Fat-rich meal**

**Pan-fried Ribeye with Stir-fried Vegetables, Avocado, and Oven-Baked French Fries**

Ingredients

Sauce: Soy sauce: 5 g, rapeseed oil: 3 g, (optional garlic), a pinch of sambal oelek, a pinch of tomato paste, 0.3 tsp freshly grated ginger, salt, pepper

Rapeseed oil: 5 g

Frozen corn kernels: 30 g

Fresh broccoli: 40 g

Carrot: 40 g

Yellow/red onion: 30 g

Avocado, as a side: 60 g

Ribeye steak, pan-fried: 75 g

French fries, frozen, 7% fat, baked in the oven: 100 g

Preparation

Mix and combine the sauce ingredients.

Slice the vegetables, stir-fry them in the oil until they are evenly soft.

Drizzle the sauce over the stir-fried vegetables.

**Supplementary Figure S1.** Trajectories for insulin, glucagon, the IGR, and glucose for each of the individuals in the study.

**Supplementary Figure S2.** Associations between the Euclidian distance to the optimal meal and diabetes duration for individuals with T2D for the carbohydrate-, fat-, fiber-, and protein-rich meals.

**Supplementary Figure S3.** Associations between the Euclidian distance to the optimal meal and BMI for the carbohydrate-, fat-, fiber-, and protein-rich meals.

**Supplementary Figure S4.** Associations between the gain in Euclidian distance to the optimal meal, relative to the carbohydrate-enriched meal, and diabetes duration for individuals with T2D for the fat-, fiber-, and protein-rich meals.
